# Fractal-Based Quantitative Collateral Assessment for Thrombectomy Candidate Selection in Acute Ischemic Stroke: A Preliminary Study

**DOI:** 10.3390/diagnostics15131590

**Published:** 2025-06-23

**Authors:** Chien-Hung Chang, Chi-Ming Ku, Tzong-Rong Ger, Wen-Piao Lin

**Affiliations:** 1 Department of Neurology, Chang Gung Memorial Hospital, Linkou Branch, College of Medicine, Chang Gung University, Taoyuan 33305, Taiwan; 2 Department and Graduate Institute of Electrical Engineering, College of Engineering, Chang Gung University, Taoyuan 33305, Taiwan; wplin@mail.cgu.edu.tw; 3 Department of Biomedical Engineering, Chung Yuan Christian University, Taoyuan 33305, Taiwan; s10125245@gmail.com (C.-M.K.); sunbow@nycu.edu.tw (T.-R.G.)

**Keywords:** acute ischemic stroke, multiphase CT angiography, collateral circulation, mechanical thrombectomy, fractal dimension

## Abstract

**Background**: Acute ischemic stroke (AIS) remains a leading cause of mortality and disability worldwide. Accurate evaluation of collateral circulation is essential for predicting outcomes following endovascular thrombectomy (EVT). However, conventional visual collateral scoring (vCS) based on multiphase CT angiography (mCTA) is limited by subjectivity and inter-observer variability. This preliminary study introduces the multiphase quantitative collateral score (mqCS), a novel imaging biomarker designed to provide an objective and reproducible assessment of both the morphological extent and temporal dynamics of collateral flow. **Methods**: In this exploratory study, 54 AIS patients treated with EVT were retrospectively analyzed. Collateral status was evaluated using both vCS (graded by two blinded neuroradiologists) and mqCS, derived from mCTA-based fractal dimension (FD) and delay indicator (DI) metrics. Logistic regression and receiver operating characteristic (ROC) analyses were performed to assess the predictive value of each scoring system for favorable 90-day functional outcomes (modified Rankin scale, mRS ≤ 2). **Results**: The mqCS was significantly associated with favorable outcomes. Patients with mqCS ≥ 0.8674 had significantly higher odds of achieving favorable outcomes (adjusted OR = 5.98, 95% CI: 1.38–25.93, *p* = 0.017; AUC = 0.80). In comparison, the visual collateral score (vCS) showed a lower adjusted predictive value (adjusted OR = 2.84, 95% CI: 1.17–6.89, *p* = 0.02; AUC = 0.79). Patients in the highest mqCS quartiles (Q3–Q4) exhibited significantly better recovery rates (69%, *p* < 0.01). **Conclusions**: This proof-of-concept study suggests that mqCS provides a potentially more objective and robust alternative to visual scoring for collateral assessment in AIS. By integrating structural and temporal characteristics, mqCS enhances outcome prediction and may inform EVT decision-making, particularly in borderline cases. These preliminary findings warrant validation in larger, prospective cohorts and support its potential integration into automated imaging platforms.

## 1. Introduction

Stroke remains a leading cause of death and disability worldwide, necessitating continuous advancements in diagnostics and treatment. Acute ischemic stroke (AIS) management has improved with intravenous tissue plasminogen activator (IVT) and endovascular thrombectomy (EVT), which enhance recovery and reduce mortality when performed within 8 h of symptom onset [1,2,3]. However, the success of EVT is highly dependent on the presence of salvageable brain tissue, particularly in anterior circulation strokes. As not all AIS patients are eligible for EVT, rapid and accurate identification of suitable candidates remains a critical priority.

Collateral circulation plays a pivotal role in influencing infarct progression and patient outcomes [4]. Effective collateral networks can mitigate ischemic damage and enhance functional recovery [5]. Computed tomography angiography (CTA) is an essential imaging modality for evaluating collateral status and predicting EVT outcomes [6]. The multiphase CTA (mCTA) provides superior temporal resolution and allows for a more comprehensive assessment of collateral circulation [7], and it has been shown to be a stronger prognostic marker for clinical and functional outcomes in AIS patients undergoing EVT [8]. Despite this advantage, current visual collateral scoring (vCS) systems based on mCTA (vCS-mCTA) remain subjective and exhibit considerable inter-rater variability [9,10], emphasizing the need for more objective and quantitative scoring approaches that can reduce observer bias in EVT candidate selection. Most existing quantitative collateral scoring (qCS) approaches [9,11] have been developed using single-phase CTA (sCTA), which provides only a static snapshot of contrast opacification and fails to capture the temporal dynamics of collateral recruitment. As a result, qCS derived from sCTA may misclassify patients with delayed but adequate collateral filling, thereby limiting their prognostic utility in borderline cases. This limitation highlights the need for time-resolved, multiparametric biomarkers that reflect both anatomical and hemodynamic features of collateral flow.

Fractal dimension (FD) is a measure of structural complexity and has been widely applied in medical imaging, especially in assessing retinal microvasculature in stroke research [12,13,14]. Lower FD values in retinal vessels have been associated with sparser vascular branching and increased cerebrovascular risk [14]. However, FD has been rarely applied to cerebral vasculature, with limited studies focusing on arteriovenous malformation (AVM) classification. Notably, FD analysis in AVM [15] has revealed that cerebral collateral vessels exhibit hierarchical branching patterns similar to those in the retina, consistent with principles of fractal geometry. These observations support the potential utility of FD as a quantitative marker of cerebral collateral status, although its application in brain collateral status remains underexplored.

To overcome this limitation, we conducted a preliminary study to develop and validate a novel multiphase quantitative collateral score (mqCS). This score integrates structural complexity, measured by fractal dimension (FD), and temporal dynamics, quantified by a delay indicator (DI), to comprehensively characterize collateral flow. This study aims to assess the prognostic value of mqCS in predicting clinical outcomes following EVT and to compare its predictive accuracy against established vCS-mCTA methods.

## 2. Materials and Methods

We retrospectively analyzed AIS patients with anterior large vessel occlusion (LVO) who presented within 8 h of symptom onset and underwent EVT at Chang Gung Memorial Hospital between April 2015 and February 2016. All patient-identifying information was anonymized according to institutional guidelines. Of the 65 patients screened, 54 were included after excluding those with prior large infarcts, inadequate imaging quality, or incomplete mCTA data due to motion artifacts or acquisition failure. Clinical risk factors, laboratory data, and imaging parameters were collected. The primary clinical outcome was defined as a favorable functional status (modified Rankin scale (mRS) ≤ 2) at 90 days.

Imaging was performed using a 320-detector CT scanner (Aquilion ONE, Canon Medical Systems, Otawara, Japan). Non-contrast CT (NCCT) was acquired to assess early ischemic changes using the Alberta Stroke Program Early CT Score (ASPECTs), followed by CTA with intravenous administration of 40 mL of iohexol (Omnipaque 300 mg/mL) at 4 mL/s. mCTA was performed in arterial, peak venous, and late venous phases, covering from the aortic arch to the cranial vertex with a slice thickness of 10–20 mm. These three-phase acquisitions enable dynamic visualization of collateral vessel filling over time, forming the foundation for both qualitative and quantitative assessment. Among qualitative methods, the Menon multiphase collateral score (vCS-mCTA) [16] is widely accepted for evaluating collateral status based on the spatial extent and temporal delay of pial vessel filling. However, its subjective nature introduces inter-observer variability, and its categorical nature limits sensitivity to subtle differences in perfusion dynamics.

To address these limitations, we developed a quantitative framework that reflects the dual-domain rationale of vCS-mCTA but applies continuous, observer-independent metrics derived from image analysis. This approach integrates both structural and temporal components of collateral physiology to provide a more reproducible alternative to visual scoring. Details of the image preprocessing algorithms, thresholding techniques, FD calculations, DI derivation, and final mqCS formulation are provided in the Supplementary Methods. These were included as Supplementary Material to avoid disrupting the main text with extensive equations and algorithmic descriptions. The overall analytical pipeline comprised five major components: (1) visual collateral scoring, (2) fractal analysis of collateral complexity, (3) DI for temporal perfusion assessment, (4) computation of the multiphase quantitative collateral score (mqCS), and (5) statistical analysis. Each of these components is described in detail below.

### 2.1. Visual Collateral Scoring

Collateral status was evaluated using the multiphase Menon visual collateral scoring system (vCS-mCTA) [16] by two board-certified neurologists blinded to clinical data. This 6-point ordinal scale (0–5) incorporates both the spatial extent and temporal delay of collateral filling. A score of 5 indicates no delay in filling and normal extent of pial vessels in the affected hemisphere relative to the asymptomatic side, whereas lower scores reflect increasing perfusion delay and diminished vessel prominence. Scores ≤ 3 are typically interpreted as representing poor collateral status and are associated with worse clinical outcomes. This vCS-mCTA framework integrates both the anatomical extent and the timing of collateral perfusion. This scoring framework formed the clinical reference standard that guided the design of our mqCS model.

### 2.2. Quantifying Collateral Complexity: Fractal Dimension Analysis

Collateral data processing was conducted using a Python 3.9-based pipeline that integrated the source code FracLac (version 2014Marb766) for ImageJ (version 1.48) and consisted of three main steps: (1) Preprocessing: images from each phase of the mCTA series were processed using adaptive thresholding techniques to enhance vessel contrast and suppress background noise artifacts. (2) FD analysis: Box-counting FD values were semi-automatically computed for both the symptomatic and contralateral hemispheres at two anatomical levels—ganglionic and supraganglionic. The analysis encompassed the proximal M1 to M2 branches within the middle cerebral artery (MCA) territory. FD ratios were calculated by dividing the FD of the symptomatic hemisphere by that of the contralateral side for each level. (3) Maximum FD ratio (MaxFD) determination: To account for delayed collateral filling, FD ratios were computed across all three mCTA phases, and the MaxFD was defined as the highest observed value. This approach highlights the phase in which vascular complexity peaks, enabling a more comprehensive assessment of structural collateral capacity while mitigating timing variability.

### 2.3. Delay Indicator: Profiling Temporal Perfusion Dynamics

To capture the temporal dynamics of collateral recruitment, we introduced the DI—a metric derived from vessel density (VD) trends across mCTA phases. For each phase, VD was calculated as the proportion of segmented vascular pixels within a standardized brain region, then normalized to generate the vessel density distribution ratio (VDDR). A linear regression was applied to VDDR values across phases to derive a slope representing perfusion timing: flatter or negative slopes indicated early-phase dominance, whereas positive slopes suggested delayed filling. The DI was defined as one minus the slope difference between hemispheres, thus reflecting the degree of temporal mismatch. Higher DI values indicate delayed but structurally preserved collateral engagement. By quantifying perfusion latency, the DI complements structural metrics, such as MaxFD, and addresses a key limitation of sCTA, which cannot capture temporal recruitment.

### 2.4. Multiphase Quantitative Collateral Score (mqCS)

The mqCS was calculated by multiplying the MaxFD and DI, thereby integrating both the spatial complexity and temporal dynamics of collateral circulation into a single composite index. This design emulates the clinical rationale of vCS-mCTA, which considers both vascular extent and filling speed, but offers a continuous, objective alternative suitable for semi-automated quantification. The reproducibility of mqCS supports its potential for integration into PACS-based or AI-driven stroke workflows.

### 2.5. Statistical Analysis

All statistical analyses were conducted using Python (version 3.10) and relevant open-source libraries. Normality of continuous variables was evaluated using the Shapiro–Wilk test. Data were summarized as mean ± standard deviation (SD) for continuous variables and as frequencies and percentages for categorical variables. Between-group comparisons were performed using the Student’s *t*-test or Mann–Whitney U test for continuous data, and the chi-square or Fisher’s exact test for categorical variables, as appropriate. The discriminative performance of the mqCS was assessed using receiver operating characteristic (ROC) analysis, and the optimal cutoff value was identified using Youden’s index. Multivariable logistic regression was used to assess the independent association between mqCS and favorable outcome (mRS ≤ 2), adjusting for age, gender, LDL, and the pre-EVT NIH Stroke Scale (pre-NIHSS) score. Model calibration was assessed using the Hosmer–Lemeshow goodness-of-fit test, and model fit was evaluated using the Akaike Information Criterion (AIC). A two-tailed *p*-value < 0.05 was considered statistically significant.

## 3. Results

The Results Section is organized into six analytic domains: cohort characteristics, associations between quantitative biomarkers and outcomes, temporal profiling of collateral recruitment, stratification performance of mqCS, concordance with functional outcomes, and multivariate predictive modeling. Detailed analyses for each aspect are presented below.

### 3.1. Clinical and Demographic Characteristics

Among 65 screened AIS patients with anterior circulation LVO who underwent EVT, 54 were included after excluding those with poor imaging quality, incomplete mCTA data, or prior large infarcts. Six patients (9.2%, 6/65) were excluded due to incomplete mCTA acquisition, particularly missing phases 2 or 3, which was primarily caused by patient agitation or suboptimal imaging quality. The cohort had a mean age of 64.84 ± 13.85 years, and 64.9% were male. Patients with favorable 90-day outcomes (mRS ≤ 2) were younger (*p* = 0.045), more likely male (*p* = 0.048), and had significantly lower pre-EVT NIHSS scores (*p* = 0.001) and LDL levels (*p* = 0.007). No significant group differences were found in occlusion laterality, treatment modality (EVT vs. EVT + IVT), or thrombectomy technique (Table 1 and Table 2).

### 3.2. Quantitative Collateral Biomarkers and Clinical Outcomes

**Figure 1:**
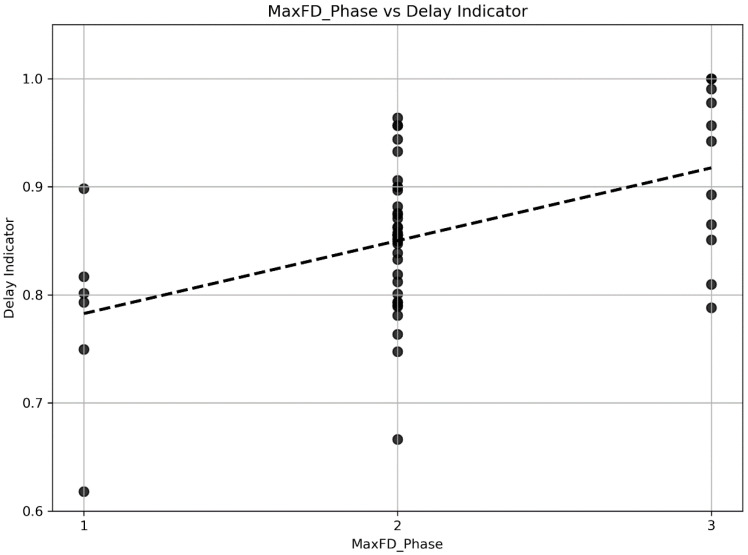
Scatter plot showing the relationship between the delay indicator (DI) and the phase in which the maximum fractal dimension (MaxFD) was observed. Each point represents a patient, with the MaxFD phase (x-axis: phase 1, 2, or 3) plotted against the corresponding DI value (y-axis). The dashed line indicates a fitted linear regression trend, suggesting a moderate positive correlation. This result supports the conceptual complementarity of temporal (DI) and structural (MaxFD) metrics in collateral assessment, with later MaxFD phases generally associated with higher DI values, reflecting delayed collateral recruitment.

Quantitative analysis revealed that patients with favorable outcomes had significantly higher mqCS values (0.825 ± 0.119 vs. 0.738 ± 0.138, *p* = 0.016; Table 2). The DI was also significantly elevated in the favorable group (*p* = 0.023), reflecting more sustained contrast progression and suggesting more favorable perfusion timing. Although the MaxFD alone showed only a statistical trend (*p* = 0.075), its integration with DI in the mqCS framework supports their complementary value. Figure 1 illustrates a moderate positive correlation between the mCTA phase in which MaxFD occurred and the DI (Spearman ρ = 0.38, *p* = 0.004), indicating that delayed structural peaks tend to align with prolonged perfusion timing. However, this association is not absolute. Several patients with MaxFD occurring in mCTA phases 1 or 2 had DI values > 0.9, suggesting delayed perfusion despite early structural expression. Conversely, some cases with MaxFD in phase 3 showed DI values < 0.75, indicating earlier perfusion despite later morphological enhancement. These heterogeneous patterns underscore the complexity of collateral dynamics and reinforce the rationale for combining MaxFD and DI into a composite index, such as mqCS.

### 3.3. Temporal Dynamics in Collateral Recruitment

**Figure 2:**
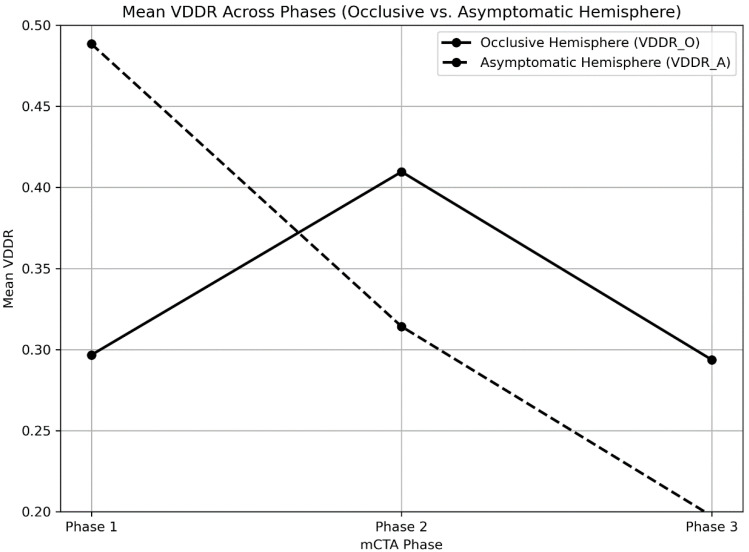
Mean vessel density distribution ratio (VDDR) across mCTA phases in the occlusive versus asymptomatic hemispheres. The VDDR represents the ratio of segmented vessel density between the occluded and contralateral hemispheres at each phase. In patients with favorable outcomes, the occluded hemisphere (solid line) shows an early peak in vessel density (phase 2), approximating the perfusion pattern of the contralateral side (dashed line), which demonstrates a progressive decline, as expected under normal physiology. In contrast, unfavorable outcome cases often exhibit delayed peaking in the occluded hemisphere, leading to greater temporal mismatch. To quantify this mismatch, a linear regression was fitted to the VDDR trajectory across phases for each hemisphere. The slope difference between the occluded and contralateral hemispheres was then used to compute the delay indicator (DI), defined as one minus this difference. A higher DI indicates more delayed collateral filling relative to the asymptomatic side. These phase-specific VDDR patterns thus provide the temporal component of the multiphase quantitative collateral score (mqCS) and are instrumental in improving prognostic stratification by capturing delayed, yet morphologically intact, collateral responses.

To further characterize perfusion timing differences, we analyzed the vessel density distribution ratio (VDDR) across the three mCTA phases for both hemispheres (Figure 2). The contralateral hemisphere demonstrated a monotonically decreasing pattern, with mean VDDR values of 0.488, 0.318, and 0.200 across phases 1 to 3, respectively. In contrast, the occluded hemisphere exhibited a delayed peak, with VDDR rising from 0.297 in phase 1 to 0.412 in phase 2 before declining to 0.295 in phase 3. This divergent temporal profile suggests a delay in peak collateral enhancement on the affected side, consistent with the pathophysiology of slow retrograde filling. The DI captures this inter-hemispheric slope difference by quantifying the extent of temporal mismatch. Patients with favorable outcomes tended to exhibit higher DI values, indicating better recruitment despite timing delays. These findings support the clinical relevance of incorporating perfusion dynamics into collateral evaluation and validate the use of DI as a temporal biomarker within the mqCS framework.

### 3.4. Stratification Performance of mqCS

**Figure 3:**
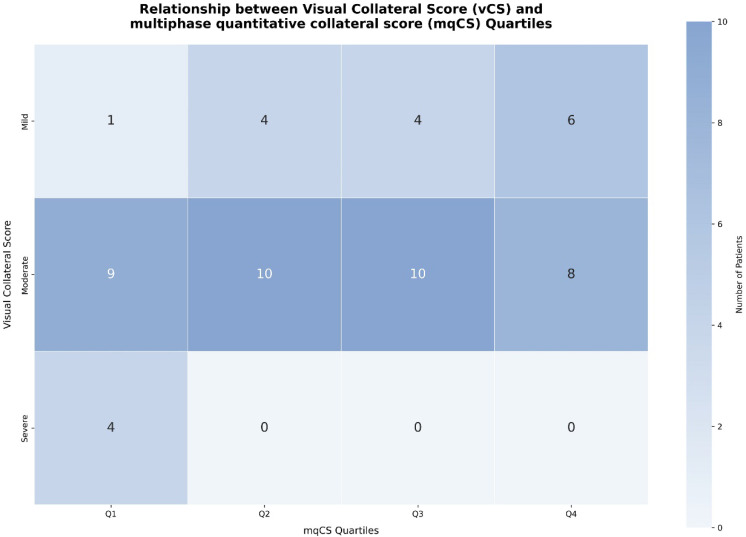
Heatmap showing the distribution of patients across visual collateral score (vCS) categories (rows) and multiphase quantitative collateral score (mqCS) quartiles (columns). The vCS was categorized as severe (0–1), moderate (2–3), or mild (4–5), while mqCS was divided into quartiles (Q1–Q4). Most patients with severe vCS were in the lowest mqCS quartile (Q1), while those with mild vCS clustered in the higher mqCS quartiles (Q3–Q4). Notably, 54% of patients with moderate vCS (score 2–3) were reclassified into higher mqCS quartiles (Q3–Q4), suggesting that mqCS may improve stratification of patients with ambiguous visual scores.

To evaluate the classification utility of mqCS, we compared its distribution across different categories of vCS. Figure 3 presents a heatmap illustrating the joint distribution of vCS and mqCS quartiles. All patients with severe vCS (scores 0–1) were classified into the lowest mqCS quartile (Q1), consistent with poor collateral status. In contrast, patients with moderate vCS scores (2–3) showed considerable heterogeneity in mqCS distribution. Approximately 48.6% of these patients were reclassified into higher mqCS quartiles (Q3–Q4), suggesting more favorable collateral physiology than visual scoring alone would indicate. This discrepancy highlights the limitations of categorical visual assessment, particularly in intermediate cases where subjective interpretation may obscure clinically relevant variations.

### 3.5. Concordance with Functional Outcomes

**Figure 4:**
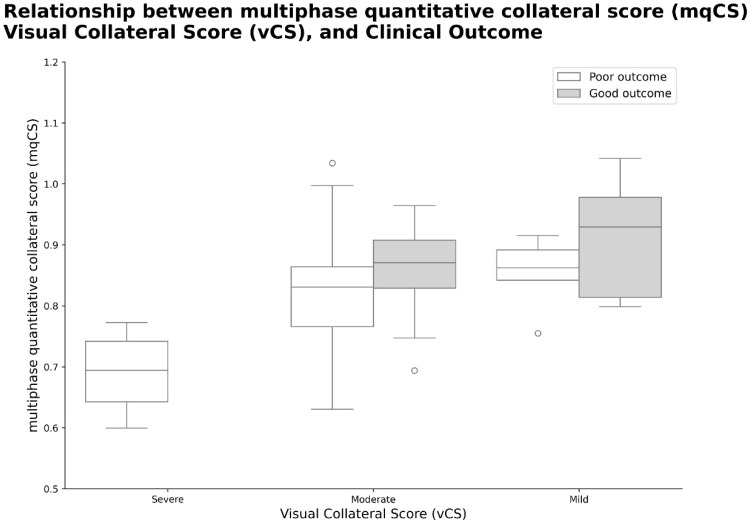
Box plot illustrating the relationship between multiphase quantitative collateral score (mqCS), visual collateral score (vCS), and clinical outcome. Patients were grouped by vCS categories—severe (0–1), moderate (2–3), and mild (4–5)—and stratified by outcome status (mRS ≤ 2 vs. >2). Within each vCS category, patients who achieved favorable outcomes tended to have higher mqCS values. Notably, in the moderate vCS group, a clear separation of mqCS values by outcome was observed, indicating the added value of mqCS in refining prognostic discrimination where visual scoring alone may be ambiguous. Circles indicate outliers, defined as values outside 1.5 times the interquartile range (IQR).

To further investigate the clinical relevance of mqCS, we evaluated its distribution across functional outcome groups (mRS ≤ 2 vs. mRS > 2) within each vCS category (Figure 4). Across all vCS strata, including severe (scores 0–1), moderate (2–3), and mild (4–5), patients with favorable outcomes consistently demonstrated higher mqCS values. Notably, within the moderate vCS category, mqCS showed clear separation between outcome groups, with patients achieving good functional outcomes exhibiting significantly higher mqCS than those with poor outcomes. This finding suggests that mqCS may capture collateral flow features not fully represented by vCS assessment, particularly in patients whose vCS fall within an equivocal range. These results support the potential utility of mqCS in improving risk stratification in patients with intermediate vCS, where prognosis tends to be more heterogeneous.

### 3.6. Multivariate Predictive Performance

To evaluate the independent prognostic utility of mqCS, we performed multivariable logistic regression adjusting for age, gender, LDL, and pre-EVT NIHSS score (Table 3). The mqCS ≥ 0.8674 was independently associated with favorable outcomes (adjusted OR = 5.98, 95% CI: 1.38–25.93, *p* = 0.017), while the vCS also retained significance (adjusted OR = 2.84, 95% CI: 1.17–6.89, *p* = 0.021). In terms of discriminative performance, the mqCS-based model achieved an AUC of 0.80, while the vCS-based model yielded an AUC of 0.79. Sensitivity was also comparable between models (65% for mqCS vs. 68% for vCS).

## 4. Discussion

This preliminary study presented the development and validation of the mqCS, a novel imaging biomarker that integrates spatial vascular complexity quantified from FD analysis and temporal perfusion dynamics assessed through a DI. In contrast to traditional vCS-mCTA, which is categorical and subject to inter-observer variability, mqCS offers a continuous, observer-independent metric for assessing collateral physiology in AIS patients. In our cohort, patients with mqCS ≥ 0.8674 were significantly more likely to achieve favorable 90-day outcomes (adjusted OR = 5.98, 95% CI: 1.38–25.93; *p* = 0.017). These findings provide preliminary evidence supporting the utility of mqCS in enhancing EVT candidate selection, particularly in borderline cases, where visual scoring yields ambiguous or insufficient prognostic insight.

**Figure 5:**
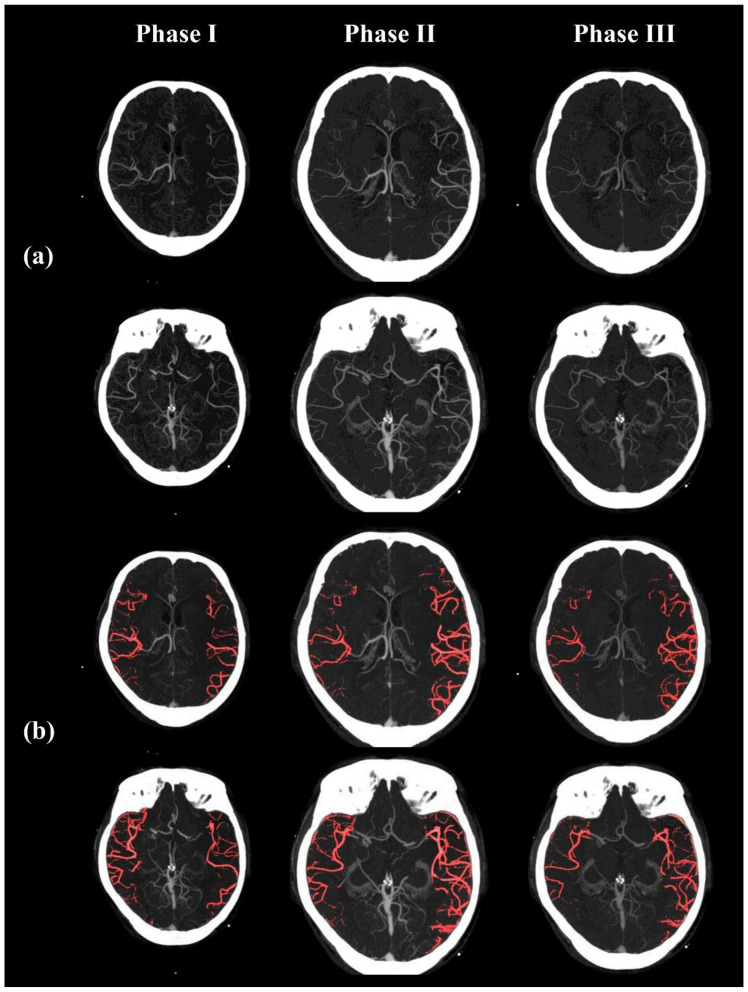
Example images of collateral vessel segmentation. It includes (**a**) original images of three phases of the ganglionic level and superganglionic level section and (**b**) an overlay of the original images and with segmented collateral vessels shown in red using the proposed method. The images are from a 73-year-old male patient with a left hemispheric large vessel occlusion. Visual collateral scoring yielded a score of 2 on single-phase CTA and 4 on multiphase CTA. Quantitative metrics were as follows: MaxFD = 1.075, delay indicator = 0.819, and mqCS = 0.880. The patient achieved complete reperfusion (TICI 3) and a favorable 90-day functional outcome (mRS = 0).

sCTA is commonly used to assess the spatial extent of collateral vessels [17], and quantitative collateral scoring (qCS) derived from sCTA has shown modest improvements over visual scoring (vCS) in outcome prediction [9,11]. These prior qCS methods, including those by Lu et al. [9] and Boers et al. [11], typically rely on static metrics, such as vessel volume, density, or region-specific enhancement. However, these approaches fail to capture temporal dynamics, such as contrast filling speed and delay, which are recognized as critical to accurate prognostication [18,19,20]. In contrast, mCTA overcomes this limitation by capturing time-resolved contrast flow. Menon et al. [16] and Souza et al. [21] have demonstrated the prognostic value of mCTA in assessing the dynamic features of collateral recruitment. Early evidence [9] suggests that qCS may offer improved predictive accuracy over vCS. However, most existing qCS [9,11] approaches have been derived from sCTA, which typically rely on collateral volume measurements and fail to quantify both the morphological extent and temporal dynamics of collateral flow. Although Boers et al. [11] showed that sCTA-derived qCS outperformed vCS in predicting AIS outcomes, the observed performance gap was modest, likely reflecting the temporal limitations of static imaging. This constraint may lead to misrepresentation of collateral physiology, particularly in patients with delayed but adequate collateral recruitment. While mCTA overcomes this limitation, its application in qCS development remains limited. To bridge this gap, we developed the mqCS, derived from mCTA. It captures both structural and temporal collateral dynamics to support candidate selection for EVT in AIS. Compared with prior qCS models, mqCS offers two key advantages. First, the inclusion of MaxFD across all three phases captures the highest degree of collateral branching, allowing identification of peak vascular complexity that single-phase methods may miss. Second, the DI provides a quantitative estimate of interhemispheric perfusion delay, complementing structural complexity with dynamic hemodynamic information. Together, these components enable mqCS to more comprehensively characterize collateral status. This is especially important in patients showing morphological–perfusion mismatch, which conventional qCS models often fail to detect. Figure 5 presents a representative case in which the patient’s vCS-sCTA score of 2 underestimated collateral adequacy, while the vCS-mCTA was higher at 4. Quantitative assessment revealed a MaxFD of 1.02 and a DI of 0.86, resulting in a high mqCS of 0.871. The patient subsequently achieved a favorable clinical outcome (mRS = 0), illustrating the potential of mqCS to resolve discordant visual assessments and more comprehensively reflect underlying collateral function.

Although higher vCS scores were generally associated with improved outcomes, the difference did not reach statistical significance (*p* = 0.08), consistent with prior studies [9], suggesting the limitations of categorical visual grading in cases of borderline or heterogeneous collateral status. This highlights the limitations of categorical vCS in certain populations. This reinforces the rationale for incorporating continuous, quantitative metrics, such as mqCS, particularly in cases with borderline or heterogeneous collateral status. In our cohort, mqCS reclassified 54% of patients with moderate visual collateral scores (vCS 2–3) into higher quantitative strata (Q3–Q4), with 27% assigned to Q4, which was associated with more favorable outcomes (Figure 3). Patients with mqCS values between 0.9 and 1.0 were more likely to achieve functional independence (mRS ≤ 2), whereas those clustered around 0.7 experienced poorer recovery, suggesting a potential association between higher mqCS and improved prognosis. Furthermore, patients in the upper mqCS quartiles (Q3–Q4) exhibited significantly higher rates of favorable 90-day outcomes compared to those in lower quartiles (*p* < 0.01; Figure 4). These findings suggest the potential of mqCS to improve stratification granularity beyond conventional vCS, particularly in cases with ambiguous or intermediate vCS scores where prognostic uncertainty is common. In multivariable models (Table 3), mqCS ≥ 0.8674 remained independently associated with favorable outcomes (adjusted OR = 5.98, 95% CI: 1.38–25.93; *p* = 0.017). Although the mqCS and vCS models demonstrated comparable AUCs (0.80 vs. 0.79), vCS exhibited slightly higher sensitivity (68% vs. 65%). Despite this, the mqCS offers additional value through its reproducibility, continuous scaling, and integration of temporal and morphological components, particularly in cases with borderline or heterogeneous collateral status, where visual grading alone may be insufficient for accurate stratification.

Although the MaxFD alone did not reach statistical significance (*p* = 0.075; Table 2), its role within the mqCS framework remains important. MaxFD reflects the structural complexity of pial collateral vessels, capturing a dimension of “morphological reserve” that is not represented by perfusion timing alone. While DI accounts for contrast arrival dynamics, MaxFD contributes complementary information about the anatomical robustness of the collateral network. Their combination enhances the sensitivity of collateral assessment, particularly in patients with discordant structural and temporal patterns. While some may question the necessity of incorporating MaxFD because DI independently predicts outcomes (*p* = 0.023), it is important to recognize that these two parameters quantify distinct and non-redundant aspects of collateral physiology. The DI reflects the temporal dynamics of perfusion, capturing how rapidly contrast arrives at the ischemic territory. However, it does not account for the anatomical extent or complexity of the collateral vasculature. In contrast, MaxFD identifies the phase in which the occluded hemisphere exhibits the highest fractal complexity, thus representing the peak structural capacity of the pial collateral network, independent of perfusion timing. Their integration within mqCS provides a more holistic assessment of collateral function by combining spatial and temporal dimensions. This integration aligns with recent studies [22] emphasizing the importance of evaluating both spatial and temporal characteristics of collateral flow in AIS. Figure 1 illustrates a moderate positive correlation between the phase of MaxFD and DI, suggesting that later structural peaks are generally associated with delayed perfusion. However, considerable inter-individual variability in collateral physiology was observed, particularly in the relationship between perfusion timing and vascular complexity. Some patients exhibited early perfusion (low DI) despite limited collateral branching (low MaxFD), while others demonstrated well-developed vascular structures (high MaxFD) with delayed contrast arrival (high DI). This divergence highlights the limitation of relying on DI alone, which may fail to detect patients with structurally intact but hemodynamically sluggish collaterals. These findings support the rationale for combining temporal and structural metrics within the mqCS framework to better account for heterogeneous collateral responses in AIS.

To derive the DI, we applied linear regression to the vessel density distribution ratios (VDDRs) across the three mCTA phases for both hemispheres. The VDDR reflects the relative temporal pattern of vessel opacification across the three mCTA phases, calculated as the ratio of vessel density on the occluded side to that on the contralateral hemisphere. In cases of prompt collateral recruitment, VDDR trajectories from both hemispheres exhibit similar slopes, indicating synchronous contrast filling. Conversely, delayed collateral engagement is characterized by a flatter VDDR slope on the occluded side, indicating a slower rate of contrast enhancement over time. The DI is defined as one minus the slope difference between hemispheres, thereby quantifying the degree of temporal mismatch. Higher DI values correspond to greater delays in collateral engagement. Figure 2 further illustrates the asymmetric VDDR trajectories observed between hemispheres: patients with favorable outcomes typically exhibit symmetric VDDR trajectories peaking in earlier phases, consistent with timely collateral flow. By capturing this temporal dimension, the DI complements structural complexity and enables mqCS to reflect both anatomical and dynamic features of collateral circulation. This dual-component integration enables mqCS not only to quantify both anatomical and temporal features of collaterals but also to enhance clinical prognostication and inform decision-making in complex or borderline EVT cases.

Our multivariable logistic regression analysis (Table 3) reinforced the prognostic value of mqCS in predicting favorable 90-day outcomes (mRS ≤ 2) following EVT. Patients with mqCS ≥ 0.8674 had significantly greater odds of favorable outcomes in both unadjusted (OR = 7.84, 95% CI: 2.30–26.65; *p* = 0.001) and adjusted models (5.98, 95% CI: 1.38–25.93; *p* = 0.017). In contrast, the adjusted OR for vCS was 2.84 (95% CI: 1.17–6.89; *p* = 0.021).

The AUCs for the mqCS and vCS models were comparable (0.80 vs. 0.79), with similar sensitivity (65% vs. 68%). While overall predictive performance was similar, mqCS offers the advantage of continuous scaling and observer independence, which may improve clinical applicability, particularly in borderline or heterogeneous cases, where visual scoring lacks sufficient granularity.

Although this study utilized mCTA and introduced a novel mqCS framework for evaluating collateral circulation, several limitations should be acknowledged. First, its retrospective design and relatively small, single-center cohort limit generalizability and preclude the establishment of causality. As this was an exploratory, proof-of-concept study, the findings are intended to be hypothesis-generating rather than confirmatory. A post hoc power analysis based on Lu et al. [9] suggested sufficient statistical power (0.92–0.99) to detect key associations; nonetheless, external validation in larger, multicenter cohorts will be essential to confirm the reproducibility and broader applicability of mqCS. A small subset of patients (n = 6) did not achieve successful recanalization following EVT. While this represents a known predictor of poor outcome, we retained these cases in the primary analysis to reflect real-world EVT variability. Future validation cohorts should consider stratifying analyses based on reperfusion success to further clarify the relationship between collateral status and functional recovery. Second, the mqCS workflow currently remains semi-automated. Manual selection of anatomical levels and region-of-interest (ROI) delineation is still required, particularly to exclude venous structures and ensure accurate assessment of vessel complexity. As noted in prior work [9], vessel segmentation remains labor-intensive and poses a barrier to streamlined stroke imaging. Although our method requires less than 90 s per case, it still depends on manual steps that introduce variability and hinder integration into time-sensitive clinical workflows. These limitations highlight the need for automation to support efficient and reproducible quantification in routine practice. The current algorithm also relies on external Python-based execution and manual DICOM file handling, further limiting clinical deployment. To address these challenges, we are actively exploring AI-based automation strategies. Specifically, convolutional neural network architectures, such as U-Net, are under evaluation for vessel segmentation and anatomical level identification. These approaches may enable fully automated detection of collateral-rich territories. Once validated, the final workflow will be integrated into PACS systems to allow real-time, reproducible, and scalable application of mqCS in acute stroke imaging. Lastly, larger prospective multicenter trials are warranted to validate the clinical utility of mqCS and confirm the robustness of the proposed cutoff threshold. In this study, the optimal mqCS cutoff value (0.8674) was derived from the same retrospective cohort without cross-validation and should be interpreted as exploratory. While external validation is crucial for broader clinical applicability, such analyses may be limited by the current modest sample size and risk of instability. Therefore, future studies involving independent cohorts are essential to corroborate these findings and establish reliable thresholds for clinical applicability. While DSA remains the gold standard for evaluating collateral circulation, its invasiveness and limited availability restrict routine use. In contrast, mqCS offers a noninvasive, time-efficient approach using mCTA to assess both structural and temporal aspects of collateral flow. Although direct comparison was not conducted in this study, future research should validate mqCS against established DSA-based grading systems to further confirm its clinical utility.

## 5. Conclusions

This preliminary study introduced mqCS, a novel image-based scoring system that integrates fractal vascular complexity and temporal perfusion dynamics to quantify collateral status in AIS patients. Compared with traditional vCS, mqCS provided a more objective, reproducible, and composite assessment of collateral physiology. The integration of both morphological and temporal features in mqCS may enhance outcome prediction and aid in EVT candidate stratification, particularly in cases with borderline or heterogeneous collateral status. Future validation in larger, multicenter cohorts and the development of fully automated workflows will be essential to facilitate its clinical integration and scalability.

## Figures and Tables

**Table 1:** Characteristics of acute ischemic stroke patients undergoing thrombectomy: comparison between favorable and unfavorable outcomes.

Variable	All, *N* = 54	Favorable (mRS ≤ 2), *N* = 24	Unfavorable (mRS > 2), *N* = 30	*p*-Value
AIS risk factor				
Age, years	64.84 ± 13.85	60.72 ± 11.07	68.16 ± 15.10	0.045 *
Gender, Male, n (%)	37 (64.9%)	20 (80.0%)	17 (54.8%)	0.048 *
Hypertension, n (%)	28 (50.0%)	11 (44.0%)	17 (54.8%)	0.420
Diabetes Mellitus, n (%)	19 (33.9%)	6 (24.0%)	13 (41.9%)	0.159
Smoking, n (%)	16 (28.6%)	4 (16.0%)	12 (38.7%)	0.061
Dyslipidemia, n (%)	9 (16.1%)	1 (4.0%)	8 (25.8%)	0.027 *
Atrial Fibrillation, n (%)	18 (32.1%)	7 (28.0%)	11 (35.5%)	0.551
Old Stroke, n (%)	8 (14.3%)	4 (16.0%)	4 (12.9%)	0.742
Gout, n (%)	4 (7.1%)	1 (4.0%)	3 (9.7%)	0.412
Clinical parameter				
Occlusion, Left, n (%)	23 (41.1%)	9 (36.0%)	14 (45.2%)	0.488
Etiology, CE, n (%)	26 (55.3%)	12 (57.1%)	14 (53.8%)	0.821
Pre-NIHSS	18.18 ± 6.87	15.00 ± 6.08	20.74 ± 6.47	0.001 **
3MRS	2.84 ± 1.99	0.88 ± 0.83	4.42 ± 0.99	<0.001 **
Laboratory values				
Cholesterol (mg/dL)	177.71 ± 44.36	166.92 ± 31.85	186.96 ± 51.58	0.105
HbA1C (%)	6.69 ± 1.549	6.430 ± 1.045	6.919 ± 1.879	0.275
LDL (mg/dL)	109.89 ± 32.70	96.75 ± 26.00	120.40 ± 34.08	0.007 **
Triglyceride (mg/dL)	105.17 ± 46.20	100.33 ± 51.41	109.03 ± 42.08	0.497
Uric Acid (mg/dL)	4.84 ± 1.61	4.82 ± 1.46	4.87 ± 1.76	0.913
Sugar (mg/dL)	113.06 ± 41.02	113.59 ± 48.58	112.56 ± 33.81	0.942
Creatine (mg/dL)	1.02 ± 0.39	0.95 ± 0.18	1.08 ± 0.49	0.217
eGFR (mL/min/1.73 m^2^)	82.015 ± 29.19	85.97 ± 23.87	78.83 ± 32.92	0.368

*: *p* < 0.05; **: *p* < 0.01. Abbreviations. AIS: acute ischemic stroke; CE: cardiac embolism; Pre-NIHSS: before thrombectomy National Institutes of Health Stroke Scale; 3MRS: 3 months modified Rankin scale; eGFR: estimated glomerular filtration rate; HbA1C: glycated hemoglobin; LDL: low-density lipoprotein.

**Table 2:** Endovascular thrombectomy (EVT) and imaging characteristics in acute ischemic stroke patients.

Variable	All, *N* = 54	Favorable (mRS ≤ 2), *N* = 24	Unfavorable (mRS > 2), *N* = 30	*p*-Value
Thrombectomy Parameters				
EVT, n (%)	34 (60.7%)	14 (56.0%)	20 (64.5%)	0.517
EVT + IVT, n (%)	22 (39.3%)	11 (44.0%)	11 (35.5%)	0.517
MCA occlusion, n (%)	24 (43.6%)	11 (44.0%)	13 (43.3%)	0.960
EVT technique				0.877
Aspiration only, n (%)	32 (59.3%)	14 (58.3%)	18 (60.0%)	
Stent retriever or combined, n (%)	22 (40.7%)	10 (41.7%)	12 (40.0%)	
TICI score				0.682
0–2a, n (%)	6 (11.1%)	2 (8.3%)	4 (13.3%)	
≥2b, n (%)	48 (88.9%)	22 (91.7%)	26 (86.7%)	
Onset-to-groin puncture time(min), mean ± SD	331.14 ± 355.21	315.12 ± 370.83	343.97 ± 345.22	0.765
Procedure duration (min), mean ± SD	61.39 ± 35.13	57.80 ± 20.17	64.32 ± 44.11	0.497
Number of passes, mean ± SD	2.11 ± 1.27	1.92 ± 1.04	2.26 ± 1.44	0.328
Imaging Biomarkers				
ASPECT score, mean ± SD	8.46 ± 1.74	8.36 ± 2.02	8.55 ± 1.50	0.690
vCS-mCTA, mean ± SD	2.80 ± 0.88	3.08 ± 0.76	2.58 ± 0.92	0.034 *
Maximum FD ratio, mean ± SD	0.99 ± 0.06	1.003 ± 0.05	0.976 ± 0.06	0.075
Delay indicator, mean ± SD	0.86 ± 0.08	0.88 ± 0.07	0.84 ± 0.08	0.023 *
mqCS, mean ± SD	0.78 ± 0.14	0.83 ± 0.12	0.74 ± 0.14	0.016 *

*: *p* < 0.05. Abbreviations. EVT: endovascular thrombectomy; IVT: intravenous thrombolysis; MCA: middle cerebral artery; TICI score: Thrombolysis in Cerebral Infarction score; ASPECT score: The Alberta Stroke Program Early CT Score; mCTA: multiphase CT angiography; FD: fractal dimension; mqCS: multiphase quantitative collateral score; vCS-mCTA: mCTA-derived visual collateral score.

**Table 3:** Multivariable logistic regression analysis predicting good outcome (mRS ≤ 2).

Variable	OR (95% CI)	*p*	OR (95% CI)	*p*
	mqCS ≥ 0.8674		Visual collateral score (vCS-CTA)	
Crude (unadjusted)				
mqCS ≥ 0.8674	7.84 (2.30–26.65)	0.001		
vCS-MCTA			2.77 (1.31–5.85)	0.008
AUC (95% CI)	0.74 (0.61–0.85)		0.71 (0.58–0.83)	
Sensitivity	0.70		0.39	
Adjusted for Covariates	Model 1 OR (95% CI)		Model 2 OR (95% CI)	
mqCS ≥ 0.8674	5.98 (1.38–25.93)			0.017 *
vCS-mCTA			2.84 (1.17–6.89)	0.021
Age	0.98 (0.94–1.02)	0.262	1.00 (0.94–1.05)	0.869
Gender (Male)	2.96 (0.88–10.02)	0.080	4.54 (0.93–22.07)	0.061
LDL	1.00 (0.98–1.02)	0.956	1.00 (0.98–1.02)	0.932
Pre-NIHSS	0.86 (0.77–0.96)	0.006	0.87 (0.76–0.99)	0.033
AUC (95% CI)	0.80 (0.68–0.91)		0.79 (0.66–0.90)	
Sensitivity	0.65		0.68	
Akaike Information Criterion (AIC)	66.10 (Model 1)		63.46 (Model 2)	

Crude and adjusted models were constructed for mqCS (≥0.8674) and vCS-mCTA. Covariates included age, gender, LDL, and pre-treatment NIHSS. Abbreviations. OR: odds ratio; CI: confidence interval; AUC: area under the receiver operating characteristic (ROC) curve; AIC: Akaike Information Criterion; mqCS: multiphase quantitative collateral score; vCS-mCTA: mCTA-derived visual collateral score; Pre-NIHSS: before thrombectomy National Institutes of Health Stroke Scale; LDL: low-density lipoprotein. Note: *: *p* < 0.05.

## Data Availability

Data supporting the findings of this study are available from the corresponding author upon reasonable request and with institutional approval.

## Supplementary Materials

The following supporting information can be downloaded at: https://www.mdpi.com/article/10.3390/diagnostics15131590/s1. Supplementary File S1: Technical description of image preprocessing, fractal dimension analysis, delay indicator calculation, and mqCS derivation.
