# Supplementary material for: Fractal-Based Quantitative Collateral Assessment for Thrombectomy Candidate Selection in Acute Ischemic Stroke: A Preliminary Study
Source: Diagnostics (Basel). 2025 Jun 23;15(13):1590. doi: 10.3390/diagnostics15131590 (PMC12249165; doi:10.3390/diagnostics15131590)
Supplement: Supplementary file 1 [file diagnostics-15-01590-s001.zip › diagnostics-3674370-supplementary.pdf]

Supplementary Materials

Image Processing and Fractal Dimension Analysis

Image Processing

In this study, collateral vessel segmentation was performed using a semi-automated method to extract vessels from 8-bit grayscale BMP images derived from DICOM files (0–450 Hounsfield units), defining regions of interest (ROIs) within the cranium. The process was divided into two key phases: brain region segmentation and collateral vessel segmentation.

Brain Region Segmentation:

The image was binarized at a grayscale value of 255 to define the cranium region. An 8-connected flood fill algorithm segmented the entire cranium, including the brain region. An XOR operation separated the cranium and brain regions, ensuring precise segmentation. To refine boundaries, pixels with grayscale values near 255 were removed using the Sobel operator applied iteratively three times.

Collateral Vessel Segmentation:

An adaptive threshold binarization method, based on the

triangle method, determined the binarization threshold from the image histogram. The process is shown in Figure S1. First, the histogram peak was normalized, and the normalized histogram (NH) was calculated as:

$$\mathrm{NH_i} = \mathrm{H_i} \times \frac{(255 - \mathrm{Gray\_level_{peak}})}{\mathrm{H_{peak}}}$$

Where $\mathrm{NH_i}$ is the normalized value for grayscale i, Hi is the pixel count at grayscale i, $\mathrm{Gray\_level}_{peak}$ is the grayscale value at the histogram peak, and H_{peak} is the count of pixels at the peak.

If multiple peaks exist, the rounded mean is used. A straight line was drawn between the peak and the highest grayscale value in the normalized histogram (Figure S1a). The Euclidean distance from this line to all grayscale values between the peak and highest grayscale was computed, and the grayscale value with the greatest distance was chosen as the adaptive threshold (Figure S1b). Finally, an AND operation was performed between the segmented brain region image and the binarized images. The clinician-delineated ROI was applied to extract the collateral vessels needed for quantitative analysis.

Fractal Dimension Analysis

The box-counting algorithm was employed to calculate the fractal dimension (FD) to quantify the morphological extent of collateral circulation in each phase. Since different starting positions in box-counting can yield varying results, we implemented four starting positions: upper left, lower left, upper right, and lower right. The average FD was calculated as follows:

$$\mathrm{FD} = \frac{\left(\mathrm{FD}_{\text{upper left}} + \mathrm{FD}_{\text{lower left}} + \mathrm{FD}_{\text{upper right}} + \mathrm{FD}_{\text{lower right}}\right)}{4}$$

where the FD when the starting position is set to p was calculated as follows:

$$\mathrm{FD_p} = \mathrm{slope}\left(\{\ln(\epsilon_k)\}_{k=min}^{max}, \{\ln\left(N(k)_p\right)\}_{k=min}^{max}\right)$$

where the slope function calculates the slope of a linear regression trendline. ϵk *represents the ratio of box size k to the background size (defined by the longer side of the smallest rectangle containing the foreground pixels), and* $N(k)p$ *is the number of boxes containing foreground pixels for a given box size k and starting position p.*

In this study, the minimum box size was set to 5, and the maximum box size was set at 45% of the background size. To prevent overestimation of FD when the morphological extent was

poor, set points were added to the image before FD calculation. These set points, based on brain region images (Figure S1C and Figure S1D), were defined by scanning in four directions (up, down, left, and right) until contact with the brain region was made. A rectangle around brain region and a midline were then defined, and the midpoint of each rectangle side was used as the set points for calculating the FD of each hemisphere.

Quantitative Collateral Scoring

In this study, FD and Vessel Density (VD) were employed as quantitative metrics to assess the morphological extent and phase delay status of collateral circulation, respectively. The average values from both the ganglionic and supraganglionic levels were used for all quantitative scores. The maximum FD ratio, calculated from mCTA images, represented the optimal morphological extent and was defined as follows:

$$\text{max FD ratio} = \frac{\text{FD}_{\text{O\_max}}}{\text{FD}_{\text{A\_max}}}$$

FD_{O_max} *is the maximum FD of the occlusive hemisphere across the three phases,*

FD_{A_max} *is the maximum FD of the asymptomatic hemisphere across the three phases.*

VD was used to quantify spatial gaps and irregularities in the

vascular network, providing insights into spatial variability and flow efficiency under ischemic conditions. To assess the phase stage of collateral recruitment, we calculated the Vessel Density Distribution Ratio (VDDR) across the three phases. The Delay Indicator was determined by comparing VDDR progression between the occlusive and asymptomatic hemispheres.

VD was defined as:

$$\mathrm{VD} = \frac{\mathrm{pixel\_number}_{\mathrm{foreground}}}{\mathrm{pixel\_number}_{\mathrm{background}}}$$

$pixel\_number_{foreground}$ is the number of collateral pixels obtained from image preprocessing, and $pixel\_number_{background}$ is the number of brain region pixels obtained from image preprocessing.

Since collateral flow changes over time, and mCTA captures three distinct phases, VDDR was used to quantify the phase stage across these phases. VDDR was defined as:

$$\mathrm{VDDRi} = \frac{\mathrm{VD_i}}{\mathrm{VD_1} + \mathrm{VD_2} + \mathrm{VD_3}}$$

Where VDDR_i represents the VDDR for phase i, and VD1, VD2, VD3 represent vessel density in phases 1, 2, and 3, respectively.

Simple linear regression was applied to the VDDR values across the three phases, with the slope of the trend line indicating the

collateral phase stage. The Delay Indicator was defined as:

$$\text{delay indicator} = 1 - (\text{slope}_{\text{O}} - \text{slope}_{\text{A}})$$

where $slope_O$ *is the trend line slope of VDDR in the occlusive hemisphere, and* $slope_A$ *is the trend line slope of VDDR in the asymptomatic hemisphere.*

Finally, the multiphase quantitative collateral score (mqCS) was calculated by combining the max FD ratio and the Delay indicator:

$$\text{mqCS} = \text{max FD ratio} \times \text{delay indicator}$$

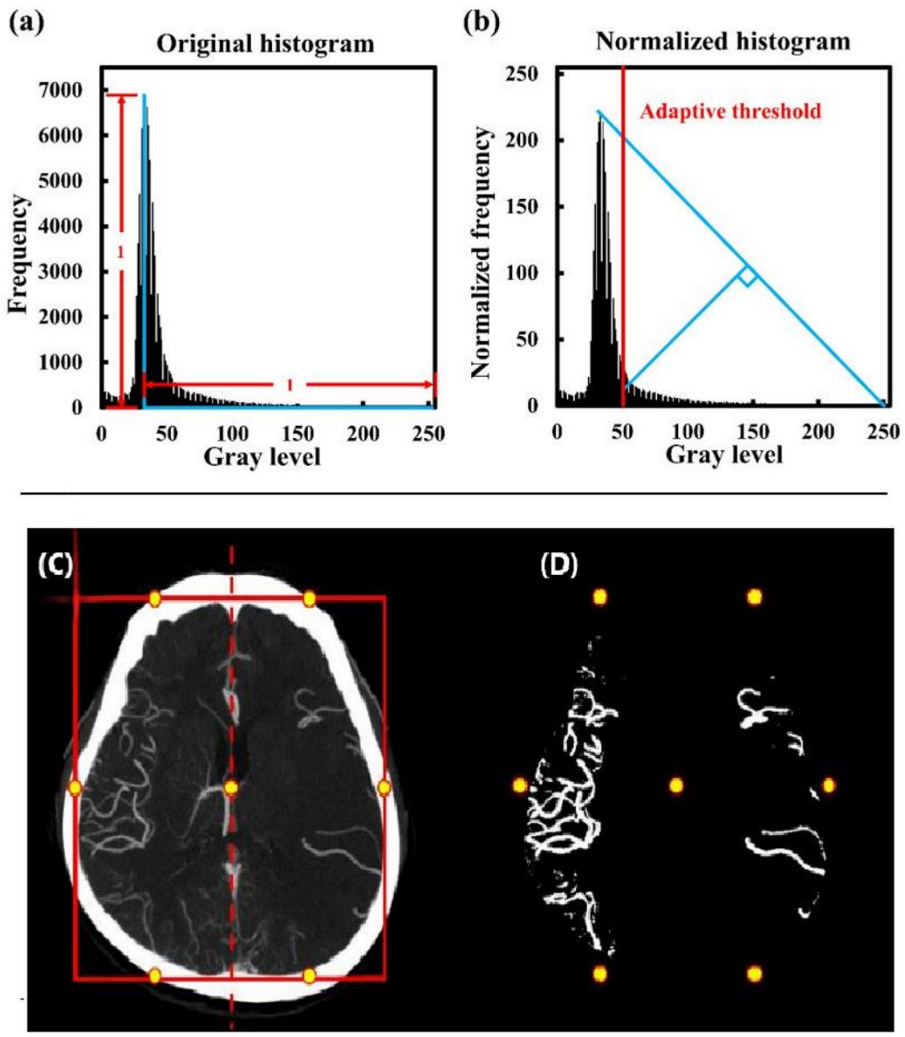

Figure S1. Schematic diagram of adaptive threshold calculation based on the triangle method. (a) Histogram of the original BMP image; (b) Normalized histogram of BMP images. Additionally, a schematic diagram of the process of adding set points is shown. (C) The locations of set points were defined based on the brain region using the scanning line (solid line) and the middle line (dashed line), and (D) these points were added to the segmented collateral vessel.
